# Psoas Abscess.—II

**Published:** 1908-02-08

**Authors:** 


### Points in Surgery.

### PSOAS ABSCESS.—II.

The treatment of a psoas abscess is at the best a disappointing business, because, although a certain number of cases are apparently cured by opening the abscess and washing it out, these must be regarded as the exception and not the rule. It follows that it must be a risky matter to give a definite prognosis in any given case. One sometimes hears the following question asked at surgical examinations: "Supposing, for the sake of argument, that you had under your care a case in which you had diagnosed the presence of a psoas abscess which was completely confined to the abdomen and did not yet show signs of pointing at any of the common situations, what would you do?" Now the answer is that a psoas abscess is rarely diagnosed as such in its early stages, even in a patient already under observation for some of the other manifestations of spinal disease, but that, if it is, no harm ensues to the patient by waiting, while surgical treatment in the nature of an extensive operation at such a time is not only harmful, but it is impracticable. Sooner or later the abscess is bound to point, and it is then that a decision must be come to as to the line of treatment.

It is obvious that the retained pus must be allowed to escape, but how best to effect this is the problem. Aspiration as a routine method has many advocates, and there are several points in its favour. But it is only a palliative measure, and unless one is prepared by experience for that eventuality one is apt to be disappointed when one finds the abscess cavity refilling. The method may be of great service when the diagnosis is in doubt. In the previous article it was said that the only condition of any practical importance which is likely to be confused with a psoas abscess is a femoral hernia. That is true. But there are certain rare diseases which occur from time to time in this situation, and are excessively misleading when they do so. One is a soft retro-peritoneal sarcoma growing from the bones of the pelvis or the structures in the posterior abdominal wall, which may cause a fluctuating swelling above or below Poupart's ligament and may exactly simulate a psoas abscess. A second disease which may lead to confusion on the right side is actinomycosis attacking the appendix. It is only within recent years that pus removed from abscess cavities has been systematically examined, and one result of this has been to throw some light on the pathology of actinomycosis. We now no longer believe that its ravages are confined to the neighbourhood of the mouth, but we know that other portions of the alimentary canal may be infected. The commonest situations are apparently the upper part of the small intestine from which the liver is involved by direct extension, and the appendix vermiformis. It is in such cases as these where the diagnosis is in doubt that aspiration is a useful procedure, because a small amount of the contents of the cavity may be removed with a trocar and submitted to microscopical examination. An incision in the first of the cases mentioned might lead to troublesome hæmorrhage, and would certainly not benefit the patient. Now the great danger of opening a psoas abscess by incision is the possibility of infecting the cavity with pyogenic micro-organisms. In favour of aspiration as a routine measure, it is argued that this rarely, if ever, occurs if the aspiration is performed with strict attention to asepsis. This argument must be allowed to be true, when one reflects how rarely a serous effusion in the pleura is converted into an empyema as the result of the insertion of a trocar. The position then may be summed up as follows. If the diagnosis is in doubt, or if the wound cannot be kept aseptic, aspiration may be justifiably performed.

But if there be no such contra-indication to operation, aspiration is not so satisfactory a method as opening the abscess by a small incision. This must, of course, be done where the abscess points. If this happens above Poupart's ligament, a small incision is made just internal to the anterior superior spine. There need be no fear of incising the peritoneum, since if the abscess is of such a size as to give evidence of its presence, this will have been pushed inwards out of the way. If, however, it points below Poupart's ligament it may be opened in the thigh. And here the incision should be made as far to the outer side as possible, because the greater the distance from the perineum the less the difficulty of securing aspesis. In a typical case a large quantity of pus will be evacuated. The question arises what to do next. Many surgeons advocate the injection of iodoform emulsion, about half an ounce of a ten per cent. solution of iodoform in glycerine, and this is described in most text-books of surgery as a routine measure. One cannot help thinking that it is one of those pieces of advice which have been transcribed from text-book to text-book without any critical investigation of its rationale being made. Iodoform when tested in the laboratory can be shown to have little or no power against the tubercle bacillus. Then how should it have any in the human body? Not only so, but the injection of iodoform into a large absorbing surface like the cavity of a psoas abscess has been known to give rise to toxic symptoms. Surely any cure must be the result of the renewed activity of the patient's natural bactericidal powers, which are restored by the removal of the caseous granulation tissue which has formed the abscess. The reasonable plan then is to irrigate the abscess with some innocuous fluid, preferably normal saline, and when this is returned clear to sew up the incision. The same argument might, it is true, be used in favour of employing a flushing gouge and scraping the wall of the cavity. But the disadvantage of this is that it is difficult to know how much tissue is being removed in such a deep cavity, and more harm may be done than good, if healthy granulation tissue is taken away. Radical operations in the lumbar region have been suggested, by which the focus of disease in the vertebral column can be attacked. But the process is generally so deep-seated and so extensive that the prospect of benefiting the patient is not proportionate to the risk involved.

